# Finite Element Evaluation of Biomimetic Porous Ti6Al4V Implants for Femoral Reconstruction: Mechanical Performance of Mono-Block and Modular Designs

**DOI:** 10.3390/biomimetics11080550

**Published:** 2026-08-03

**Authors:** Antonio de Nigris, Joaquin Daud, Donato Monopoli, Luigi Ambrosone

**Affiliations:** 1 Department of Medicine and Health Sciences “V. Tiberio”, University of Molise, Via De Sanctis, 86100 Campobasso, Italy; ambroson@unimol.it; 2 Department of Biomedical Engineering, Instituto Tecnologico de Canarias, C/ Lugar la Punta s/n, Pozo Izquierdo, 35119 Santa Lucía de Tirajana, Spaindmonopoli@itccanarias.org (D.M.); 3 OSTEOBIONIX PS, S. L., C/ Lugar la Punta s/n, Pozo Izquierdo, 35119 Santa Lucía de Tirajana, Spain

**Keywords:** porous implant, mono-block implant, modular implant, Von Mises stress, equivalent strain, finite element analysis, femur reconstruction

## Abstract

Two design solutions such as modular and mono-block Ti6Al4V porous implants for femoral defect repair were implemented and compared. Static stress analysis on each model was performed via finite element analysis to investigate potential critical elements that might cause system failure under physiological loads. Prior to calculations, a mesh convergence study was realized by varying the minimum element sizes. The entire bone–prosthetic system was modeled, and design optimization was performed. For mono-block implants, a less stressed configuration was found by changing the plate design. Comparison of the maximum Von Mises stress $\sigma_{max}$ and equivalent strain $\varepsilon_{eq}$ between the models allowed for an understanding of the distribution of the loads and identify areas with critical stress concentration. The modular implant appeared to be highly solicited with stress shielding on epiphyses due to enhanced rigidity at the metal/bone interface. Finally, a study of the deformation on cancellous and cortical bone suggested that a more elastic junction with balanced strain delivery to the bone might improve tissue regeneration when using a mono-block implant.

## 1. Introduction

Aggressive malignant bone tumors are most commonly treated through surgical resection followed by anatomical reconstruction with prosthetic systems [1,2]. Despite decades of progress in orthopaedic oncology, the restoration of extensive diaphyseal femoral segmental defects continues to represent a formidable engineering challenge. The clinical imperative is twofold: to provide the limb with an immediate recovery of load-bearing capacity, while simultaneously fostering the long-term regeneration of functional bone tissue. It is well established that iso-elastic implants, when compared with native bone, can deliver mechanical cues to cancellous tissue that trigger remodeling processes [3,4]. The skeletal system, however, is not a passive structure but rather a highly dynamic biological environment that continuously adapts to mechanical stimuli. When stresses at the bone–implant interface are balanced, they can drive optimal cellular turnover and new bone formation; conversely, excessive or insufficient loading may induce resorption and compromise implant integration. In this context, stress shielding remains one of the most critical limitations, often leading to premature failure of prosthetic reconstructions. Modern engineering approaches therefore seek to mitigate stress shielding while ensuring adequate structural stability. Biocompatible metallic materials, particularly titanium and its alloys such as Ti6Al4V, have emerged as gold standards, owing to their exceptional mechanical performance and compatibility with host tissues [5]. Furthermore, additive manufacturing has enabled the design of porous scaffolds with tunable architecture, capable of mimicking the anisotropic behavior of bone and enhancing osseointegration [6].

In detail, high interconnected porosity (>90%) was proven to be a key feature of durable implants because it confers to the prosthetics adequate elasticity and flexibility, which can be tuned by optimizing design parameters such as pore diameters, geometry, and orientation, as well as interconnection size and spacing [7,8]. An interesting geometry is represented by the gyroid porosity, whose continuous curvature leads to a lightweight and elastic structure with notable resistance to fatigue [9,10,11]. As a result, these features make the gyroid scaffold a perfect match between artificial and biologic matrices, providing osteoconductance and inducing osteogenesis [12,13,14]. Thus, the newly formed endochondral bone tissue forming in the interconnected interstices is highly vascularized and does not present fibrotic tissue that would prejudice the implant stability. Therefore, progressive formation of consistent bone callus reinforces the defect and preserves the elasticity. This kind of osteointegration, led by the physiological elasticity of the construct, is favored by second intention regenerating a persistent and abundant bone tissue. On the other hand, a more rigid fixation with dense connecting elements would enforce stress shielding, resulting in narrow and precarious intra-membranous callus. In this light, an ideal prosthetic system should be engineered to be exceptionally elastic and flexible, relying on interconnected high porosity to induce formation of osteochondral bone in the defect. Finally, the ideal implant should involve as few dense elements as possible that could increase stiffness and overload the fragile bone segments, disfavoring the mechanical stability.

On these grounds, both mono-block (MBI) and modular (MI) implants have been conceptualized. State-of-the-art solutions typically involve a porous Ti6Al4V scaffold anchored to the epiphyseal extremities through fixation plates [15], creating a mechanically continuous and flexible construct. Such designs are intended not merely to restore lost anatomy, but to harmonize the mechanical environment of the bone–implant system, thereby promoting both stability and biological integration. The design of MI simplifies the surgical procedures because it requires minimal distractions, which reduces operation time and decreases the exposure of the open wound. However, MI presents short-term complications [16] since they comprise critical pre-tensioned elements and fleeting contact surfaces. On the other hand, MBI, even though they require wider and arduous distractions, do not present internal micro-movements, and failure does not depend on rupture of linking elements. Finite element simulations for in silico mechanical testing of prosthetic systems in orthopaedic biomechanics emerged as a powerful tool to predict the behavior, under severe operating conditions, of biological tissues [17] or implants components, from single parts to complex assemblies [18,19]. The virtual quasi-static load transfer of joint modules in dynamic systems became routine analysis in product development because they can offer a prediction of fractures and internal interactions without actually setting up demanding and expensive laboratory experiences. Modern designing strategies, such as the 3D model reconstruction of musculoskeletal districts from computer tomography (CT) scans or magnetic resonance imaging (MRI) overcame the excessive simplification of the model’s geometry and boundary conditions, making the simulation process capable of predicting with sufficient confidence the mechanical stability of the devices. Therefore, assuming for biological volumes or porous structures a more simple geometry with experimental values of mechanical properties considerably reduces computational costs while ensuring robust results. The aim of the study was to optimize the design of reconstructive custom implants in order to avoid the use of critical elements susceptible to breakage and at the same time encourage bone regeneration. Preliminary simulations were launched to evaluate the most advantageous stress distribution in MIs and analyse the effect of converting the design into MBIs. Investigation of maximum Von Mises stress and equivalent strain through quasi-static simulations in both models allowed for a highlight of critical sections and uneven distributions, offering a deep understanding of internal settlement and repercussions on bone loading. The diverse potentialities in bone regeneration, flanked by mechanical stability and reliability, made it possible to identify the most suitable orthopaedic solution for bone remodeling and load bearing.

Previous studies have investigated modular implants, focusing on the clinical point of view [16,20] or through finite element analyses [2,21,22] comparing alternative fixation strategies. Nevertheless, these works essentially assess modular design or local component optimization, rather than directly comparing patient-specific modular and mono-block porous implants under identical boundary conditions. In contrast, the present study evaluates two reconstruction philosophies for the same femoral defect, while maintaining the same finite element framework, involving the anatomical model, material properties, loading conditions and contact behavior among components. Furthermore, beyond stress analysis on titanium implants, the strain distribution within cortical and cancellous bone is examined to discuss the potential implications for stress shielding and bone regeneration. By extending the design assessment to incorporate mechanobiological strain patterns, these comprehensive, full-scale bone–implant simulations deliver a robust identification of the superior prosthetic configuration: one that successfully mitigates stress shielding while providing the vital mechanical cues required for osteogenesis and long-term implant survival.

## 2. Methods

### 2.1. Custom-Made Implants Design

Firstly, a conventional modular solution was designed to reconstruct a femoral segmentary defect. Then, a mono-block strategy was assessed by computations in order to understand the resulting stress and strain re-distribution. Finally, a design optimization process was pursued in order to minimize the risk of implant failure under a static load.

#### Bone Reconstruction and Prosthetics Design

The femur and defect were de-novo designed on realistic anatomical models, simplified but representative, and used for virtual planning of surgical steps as well as bone and surrounding tissues resection within a safety margin. The bone defect was, therefore, reconstructed by a custom Ti6Al4V implant. The digital bones alignment and virtual tumor resection were performed using Fusion (Autodesk, v. 2601.0.90). In order to recreate the bone defect (146 mm) both a modular implant and a mono-block implant were designed.

**Figure 1:**
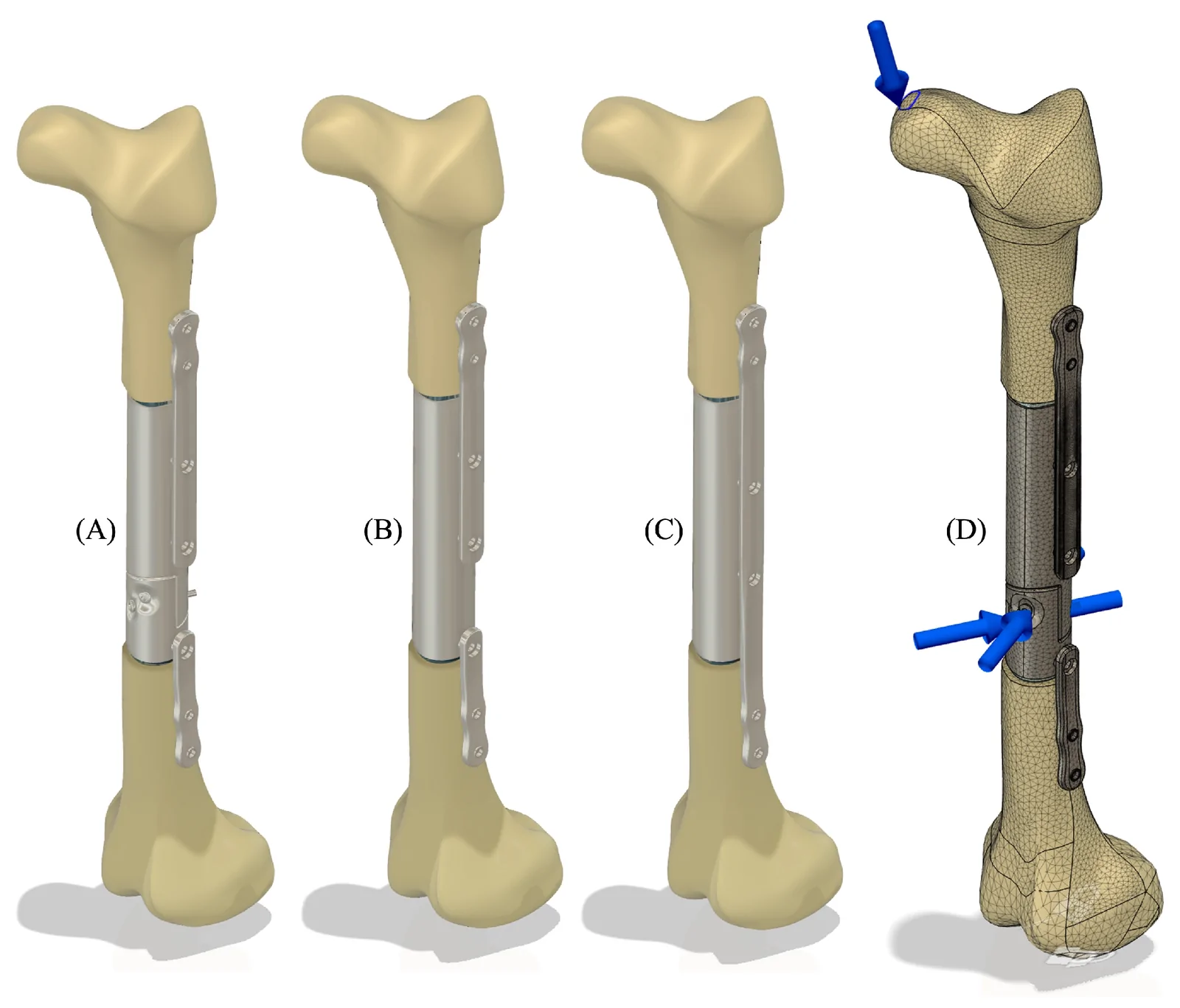
Perspective views of designed implants with simplified screws: (**A**) Modular implant, (**B**) Mono-block implant No. 1, (**C**) Mono-block implant No. 2. The porous bodies (light-green) were designed as continuous structures with porous titanium mechanical properties. (**D**) 3D model of the simulated femur–implant systems with overlapping finite elements finest mesh, constraints, and loads displayed as vectors.

*Modular implant.* The MI consisted of proximal and distal segments joined by two M4 × 35 mm linking screws (No. 1, proximal; No. 2, distal). The proximal segment was secured to the proximal-lateral cortical bone with a dense plate contoured to the surface and fixed using two self-tapping locking screws. Both segments were additionally anchored to the distal-lateral cortical bone with a dense plate and four fully threaded 3.5 × 30 mm locking screws, numbered 1–4 from proximal to distal. In the simulation model, the assembly included proximal and distal bone portions, prosthetic modules, and two screw sets (bone-linked and prosthetic-linked). Each segment was stabilized intra-medullary with a porous stem (80 mm) connected to a defect-filling porous structure, externally coated with a dense titanium shell for mechanical stability. The contacting surfaces of the proximal and distal modules were made of dense titanium alloy to allow threading and withstand screw pretension. The 3D model of the MI implant is shown in Figure 1A.

*Mono-block implants*. The starting MBI implant, henceforth referred to as MBI-1, consisted of a single Ti6Al4V ELI 23 (extra low interstitial and high purity) outer shell (0.80 mm thick) with a porous core. This design eliminated the need for connecting elements such as linking screws. Intra-medullary stabilization and fixation plates were kept identical to the MI (Figure 1B). Subsequently, a second MBI design was conceived by re-engineering the plate fixation. With the aim of reducing the stress field on the connecting bridges and on the intra-medullary stems, a single proximal-distal plate was used (Figure 1B). This system is henceforth referred to as MBI-2.

### 2.2. Finite Element Analysis

The mechanical behavior of the prosthetic systems under static loading was investigated through finite element method (FEM) simulations. Numerical analyses were performed in Autodesk Fusion 360 (v. 2025.2) using the Nastran solver, selected for its practical graphical user interface (GUI) in managing complex assemblies with multiple bodies and materials. Fusion’s contact management and meshing tools are particularly efficient for handling large numbers of contacting curved surfaces and allow for detailed analysis of screw interactions through section analysis. Contact selection is a critical aspect of prosthetics simulations and strongly affect the results [23]. The 3D-printed prosthetic geometries were modeled with their actual articulated features, except for the screws’ thread, without simplifications to minimize approximation in stress distribution and provide reliable insights into the potential failure of critical linking components. The software’s variety of contact types enabled realistic boundary condition modeling. For this study, separation, bonded, and rough contact types were applied, as described in the following sections.

#### 2.2.1. Materials Selection and Properties

The bone structures were modeled by simplifying the complex arrangement of bone layers and tissues into two distinct regions: the inner volume, represented as a bulk material with spongy bone properties, and the outer thick shell, characterized by cortical bone properties. Bone tissue properties are inherently heterogeneous and strongly influenced by the patient’s health condition. The bone health properties used in this study were taken from references [24,25,26,27,28,29]. To account for inter-subject biological variability, the inherent heterogeneity of the bone cell–matrix microenvironment, and the limited availability of patient-specific measurements in the literature, bone tissues were assumed to be isotropic. Although this assumption is expected to influence absolute simulation accuracy, it remains highly effective for developing a generalized biomechanical model focused on evaluating structural interactions and ensuring an unbiased comparative assessment of load transfer. Conversely, to accurately predict localized stress distributions within the mechanically loaded bone–implant system, a more comprehensive characterization of bone anisotropy would be required.

The equivalent Young’s modulus of the porous Ti6Al4V implant was chosen from previous experimental investigation of the mechanical response of 3D printed porous scaffolds [12,30]. Table 1 and Table 2 summarize the properties of the materials used and their assignments in the designed models.

#### 2.2.2. Model Mesh

In order to obtain realistic and reliable results, the selection of contacts between the component surface sets is crucial. The outcome of the simulation is strongly influenced by the mechanical interactions among the components in the assembly. To avoid over-constraining the prosthetic system and to allow for correct sliding between surfaces, all dense titanium surfaces in contact were set as rough contacts. The calculation implemented by Fusion treats separate bodies as capable of sliding with a default friction coefficient of 0.30, while rough contacts are assigned a higher friction coefficient. Conversely, all prosthetic fixation elements to the bone, including both cortical and cancellous surfaces, were set as separated to ensure realistic mechanical interaction. A concise yet detailed list of contact types and settings is reported in Table 3.

Regarding the contact formulation, all interacting surfaces were modeled using a symmetric penetration normal behavior to enforce non-interfering displacement of nodes across contacting components. Both *rough* and *frictionless* (separate) contact types were implemented to accommodate sliding conditions under varying frictional regimes. Specifically, the frictionless separation formulation allows unconstrained sliding between surfaces, whereas the rough formulation prevents tangential slippage by assuming a near-infinite friction coefficient. The frictionless separation type was strategically selected to capture the micro-movements at the bone–titanium interface, which are critical, as they govern tissue damage and micro-crack propagation that could ultimately trigger implant failure. Conversely, the rough contact condition was strictly applied to the modular implant (MI) assembly, where the design relies on the geometric interlocking of proximal and distal segments secured via tensioning screws. Lastly, a tied/bonded condition was assigned to the interface between trabecular and cortical bone tissues, reflecting the natural continuity of the biological matrix and the smooth physiological transition between trabeculae and osteons. A similar rationale was extended to the interfaces between porous and dense titanium components, assuming an additive manufacturing or fabrication process that ensures structural cohesion within the monolithic piece. Finally, the linking screws between dense titanium modules required particular attention due to their design. Specifically, since only half of the body of the linking screws has threads, its simplified cylindrical body was divided into two portions with different contact types. The section screwed into the dense titanium module was bonded, while the smooth surfaces were set as separate. Both assemblies were discretized using model-based finite element meshes. The final model considered for the discussion of results was selected after a mesh-independence study performed on the more complex geometry. The properties of the analyzed model are reported in Table 4. Specifically, tetrahedral elements were generated, and their dimensions were scaled for each part to ensure better sampling of the volumes and to reduce the spatial extent required for interpolating results between nodes.

#### 2.2.3. Boundary Conditions

*Constraints.* The assembly was fully fixed by constraining the bottom faces of the distal cortical bone (anatomically connected to the tibial plate), preventing displacement or rotation along all axes. Degrees of freedom were automatically set by the defined contact interactions.

*Loads.* The model simulated the prosthesis under severe loading conditions. A total load of 1.60 kN (twice the body weight of an average 80 kg male) was applied to the femoral head, distributed over a 10 mm radius area and oriented at −23° ($\hat{x}$), −5° ($\hat{y}$), and −25° ($\hat{z}$), to reproduce the acetabulum–femur load transfer. Muscular and tendinous reactions were simplified as additional superimposed loads. The effect of the linking screws was also modeled. Screw pretension after tightening was represented by axial forces acting on the module contact faces, screw head, and threads (1.00 kN on each face), following the selected contact definitions.

## 3. Results and Discussion

A static stress simulation was performed in order to evaluate the stress distribution in the whole assembly under static conditions. The aim is to assess whether mechanical discontinuities and connections would affect the stability of prosthetic system and expose the patient to undesired risk. Hence, relevant mechanical parameters resulting from the computation were investigated, such as maximum Von Mises stress and maximum equivalent strain. Von Mises stress is a physical quantity which combines the components of the stress tensor in the 3D space for the material element. For isotropic materials and in the linear-elastic range it is calculated in its general form and using principal stresses [31,32]:

(1) $$\sigma_{v}=\sqrt{\frac{1}{2}\left[{\left(\sigma_{11}-\sigma_{22}\right)}^{2}+{\left(\sigma_{22}-\sigma_{33}\right)}^{2}+{\left(\sigma_{33}-\sigma_{11}\right)}^{2}\right]+3\left(\sigma_{12}^{2}+\sigma_{23}^{2}+\sigma_{31}^{2}\right)}$$

where $\sigma_{ij}$ is the component of the stress tensor which physically acts on the face orthogonal to the $\hat{i}$ principal axis, along the $\hat{j}$ principal axis on an infinitesimal cubic volume element into the analyzed body, having i,j chosen among 1,2 and 3 principal axes. The Von Mises yield failure criterion states that the plasticity occurs when $\sigma_{v}$ > $\sigma_{y}$. Analogously, the equivalent strain is defined by the equation

(2) $$\varepsilon_{eq}=\sqrt{\frac{2}{3}\left[{\left(\varepsilon_{11}-\varepsilon_{22}\right)}^{2}+{\left(\varepsilon_{22}-\varepsilon_{33}\right)}^{2}+{\left(\varepsilon_{33}-\varepsilon_{11}\right)}^{2}\right]}$$

with $\varepsilon_{ii}$ having the strain along the $\hat{i}$ axis.

### 3.1. Convergence of the Mesh

**Figure 2:**
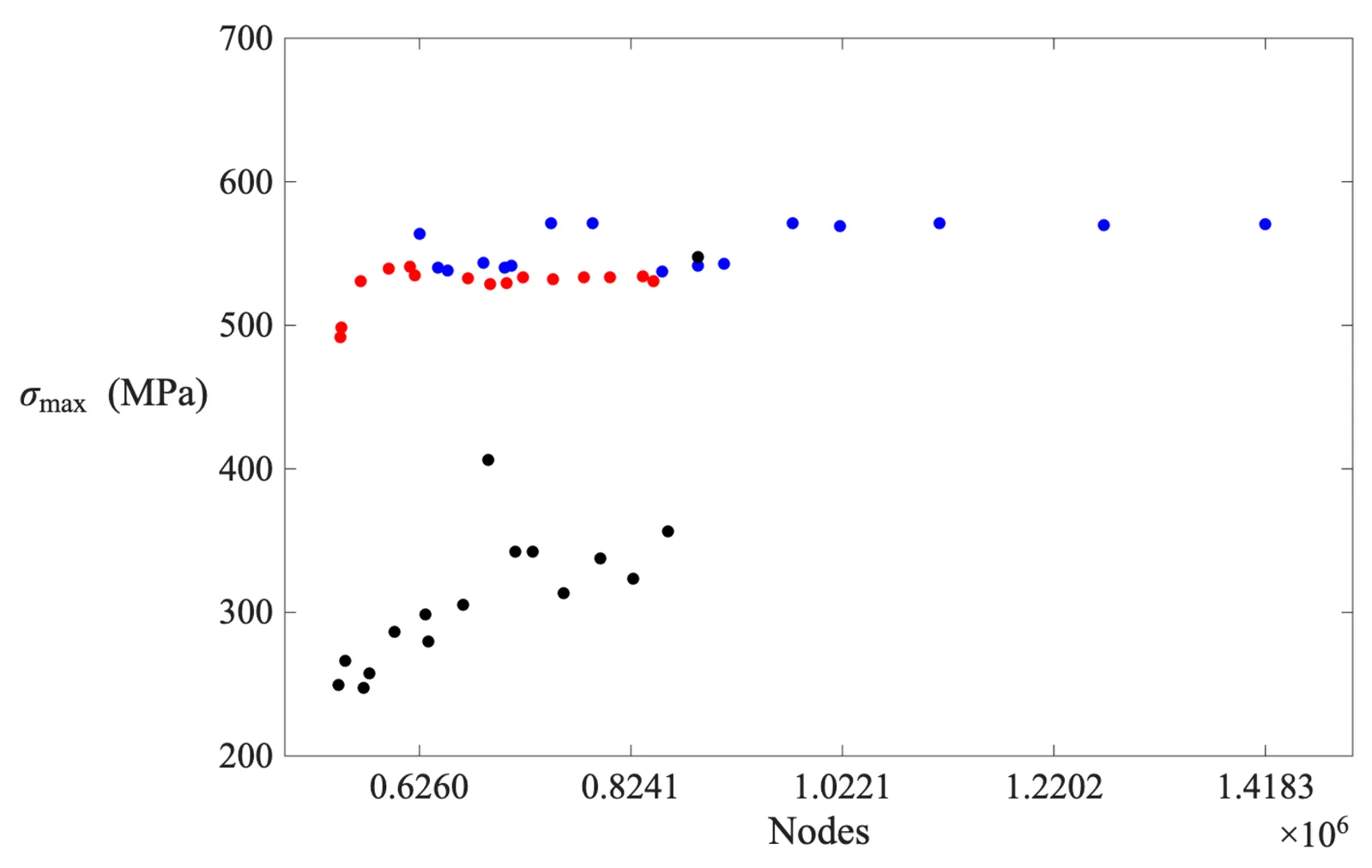
Mesh convergence study reporting maximum Von Mises stress on the distal plate bridge by varying the minimum element size from 20% to 5%, with fixed 3% average element size: MI (blue dots), MBI-1 (red dots), MBI-2 (black dots).

To avoid potential influences of mesh resolution on load distribution within the assembly, a convergence study was carried out on the complex MI starting geometry. Simulations were performed with varying levels of mesh refinement, ranging from a coarse wireframe to a fine mesh with smaller elements. To balance computational cost with solution accuracy, a step-wise mesh refinement strategy was adopted. In detail, the average element size was set to 3%, considered the minimum accessible on the software. Meshes with an average element size below 3% were computationally prohibitive and were therefore excluded. In the second step, with the average element size fixed at 3%, further refinement was applied by reducing the minimum element size from 20% to 5% in 1% increments. Element sizes below 5% were again deemed impractical. Figure 2 presents the maximum Von Mises stress on the bridge connecting the distal plate to the solid shell in the MI, MBI-1, and MBI-2 designs, since it is identified as the most critical component of the assembly. Stress is reported as a function of the average element size. To better assess the effect of mesh refinement, the relative error in Von Mises stress was defined as follows:

(3) $$E_{rel}\left(\%\right)=\frac{|\sigma_{max}^{i}-\sigma_{max}^{0}|}{\sigma_{max}^{0}}\cdot 100$$

where $\sigma_{max}$ denotes the peak stress obtained using a mesh with a minimum element size of *i* %, and $\sigma_{max}^{0}$ corresponds to the peak stress from the finest feasible mesh.

In the MI simulations, the computed relative error on the bridge is always below 5% and reaching ∼0% after $1\times 10^{6}$ nodes, while for MBI-1 the error drops to zero after a few cycles, and in the MBI-2 simulations, error follows a linear decrease. Therefore, the mesh refinement study shows that a 3% average element size with a 5% minimum element size wireframe is suitable for further refinement.

It is worth emphasizing that while the numerical stability of the present findings has been rigorously verified, this convergence does not inherently guarantee the physical accuracy of the computational model. A comprehensive experimental validation of the bone–prosthesis assembly would necessitate extensive mechanical testing on cadaveric femur segments, leveraging a statistically robust dataset to effectively mitigate the confounding effects of inter-specimen variability.

### 3.2. Model Comparison

**Figure 3:**
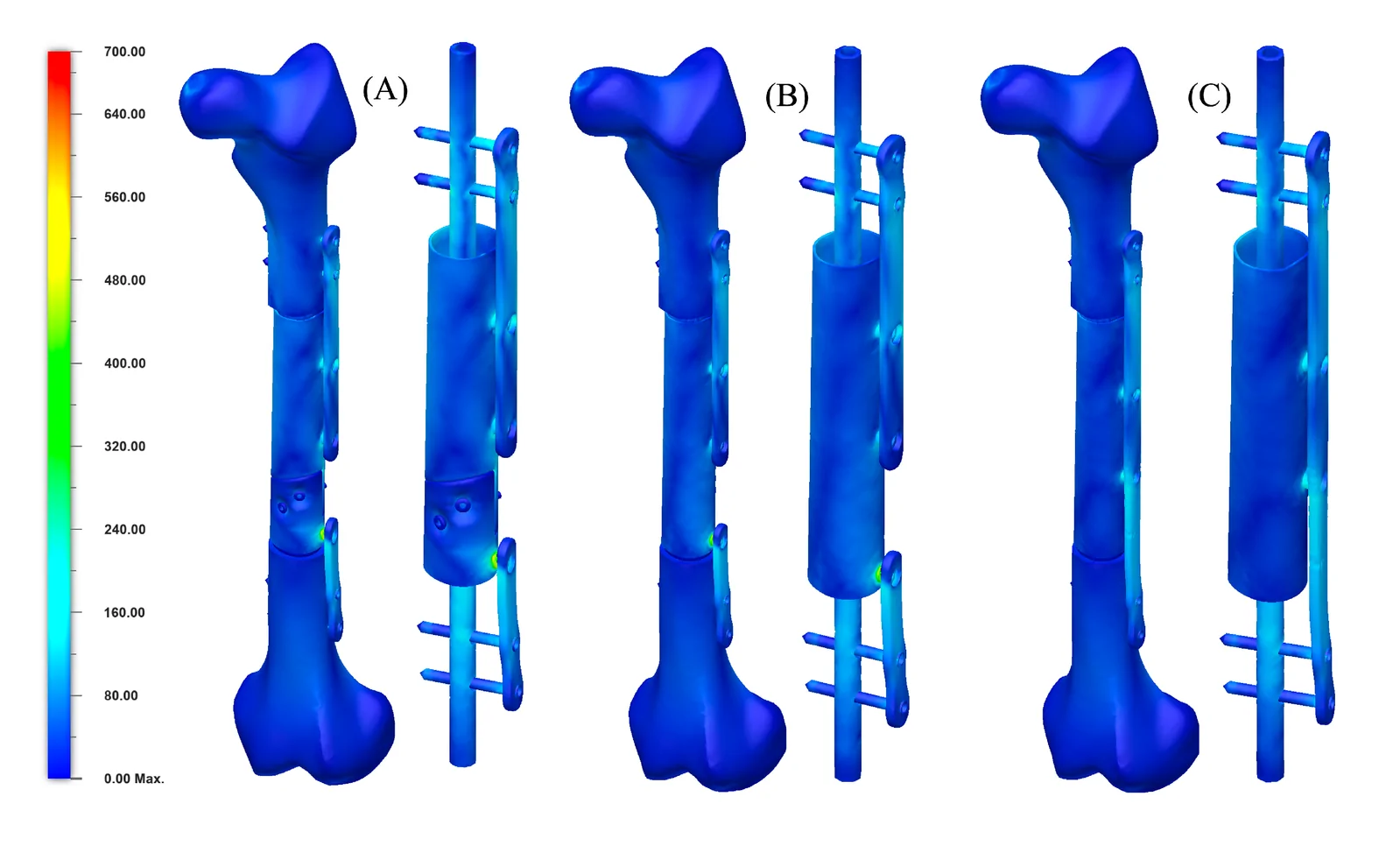
Von Mises stress $\sigma_{v}$ results. Perspective view of stress distribution on the whole bone–implant system alongside a detail on dense prosthetics: (**A**) MI, (**B**) MBI-1, and (**C**) MBI-2 implants. The scale-bar was truncated at 400 MPa to enhance critical areas: peak values are indicated by arrows.

*Titanium implants.* Figure 2 compares the maximum Von Mises stress between the MI and MBIs models. In the MI, stress concentrates in the distal and proximal screw–stem fixation region due to local rigidity and inside the plates, which are internally bent by load-bearing tension (Figure 3).

**Figure 4:**
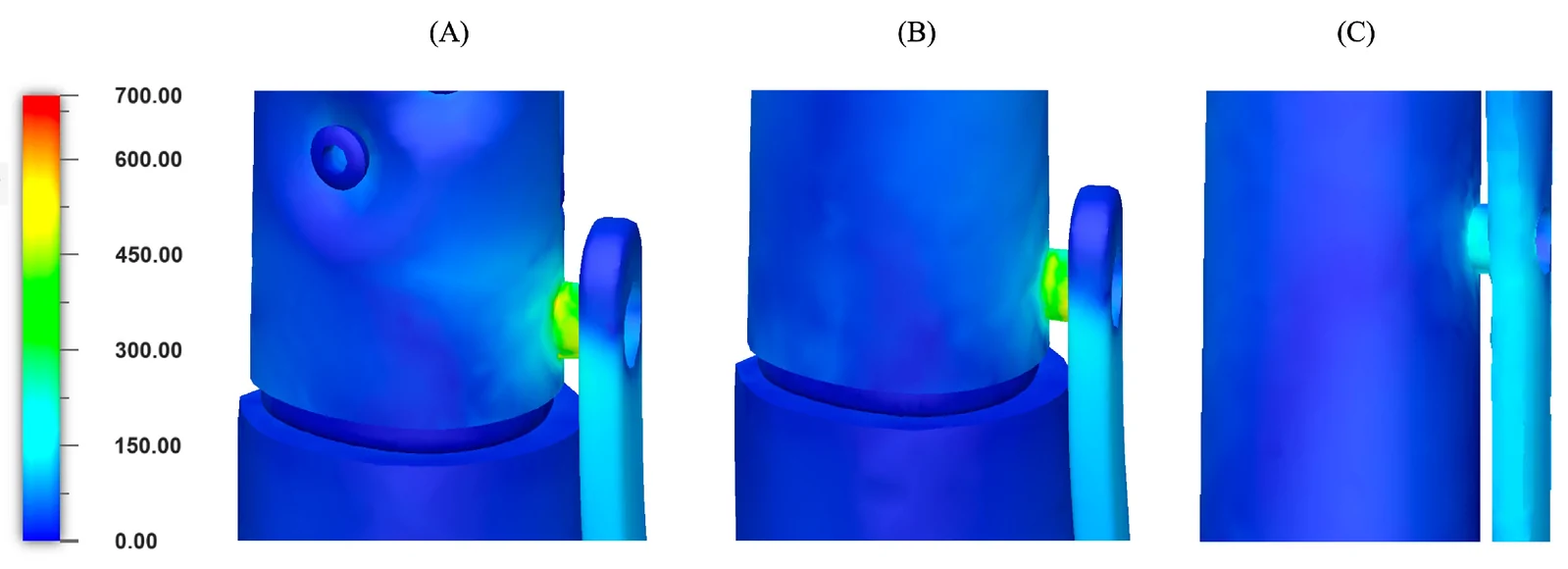
Comparison of maximum stress on the bridge connecting the distal plate to the dense shell of the implants: in the MI (**A**), MBI-1 (**B**), and MBI-2 (**C**).

In the MI, the most critical volume is near the bridge connecting the implant cylinder to the distal plates, due to its proximity to the bottom constraint, a small cross-section (38.48 ${\mathrm{mm}}^{2}$), and exposure to bending (lever arm 245 mm). Figure 4 compares the heatmaps of stress concentration on the bridge. It is evident that, in the MI model, the connecting segment is intensely loaded, with maximum stress reaching 570.42 MPa. MBI-1, due to the lack of linking screws and local mechanical discontinuities, depletes peak loading, resulting in a lower maximum stress of 560.45 MPa. In the third model, the proximal and distal plates with single-bridges were replaced with a wider single plate linked to the solid prosthetic cylinder through a couple of more symmetrically balanced bridges. In all cases, the maximum strain remains below 0.90%. Table 5 highlights the load-weakening effect on the bridges, with maximum stress diminishing to 547.58 MPa. This ensures increased mechanical stability and resistance, associated with a minimized risk of breakage.

**Figure 5:**
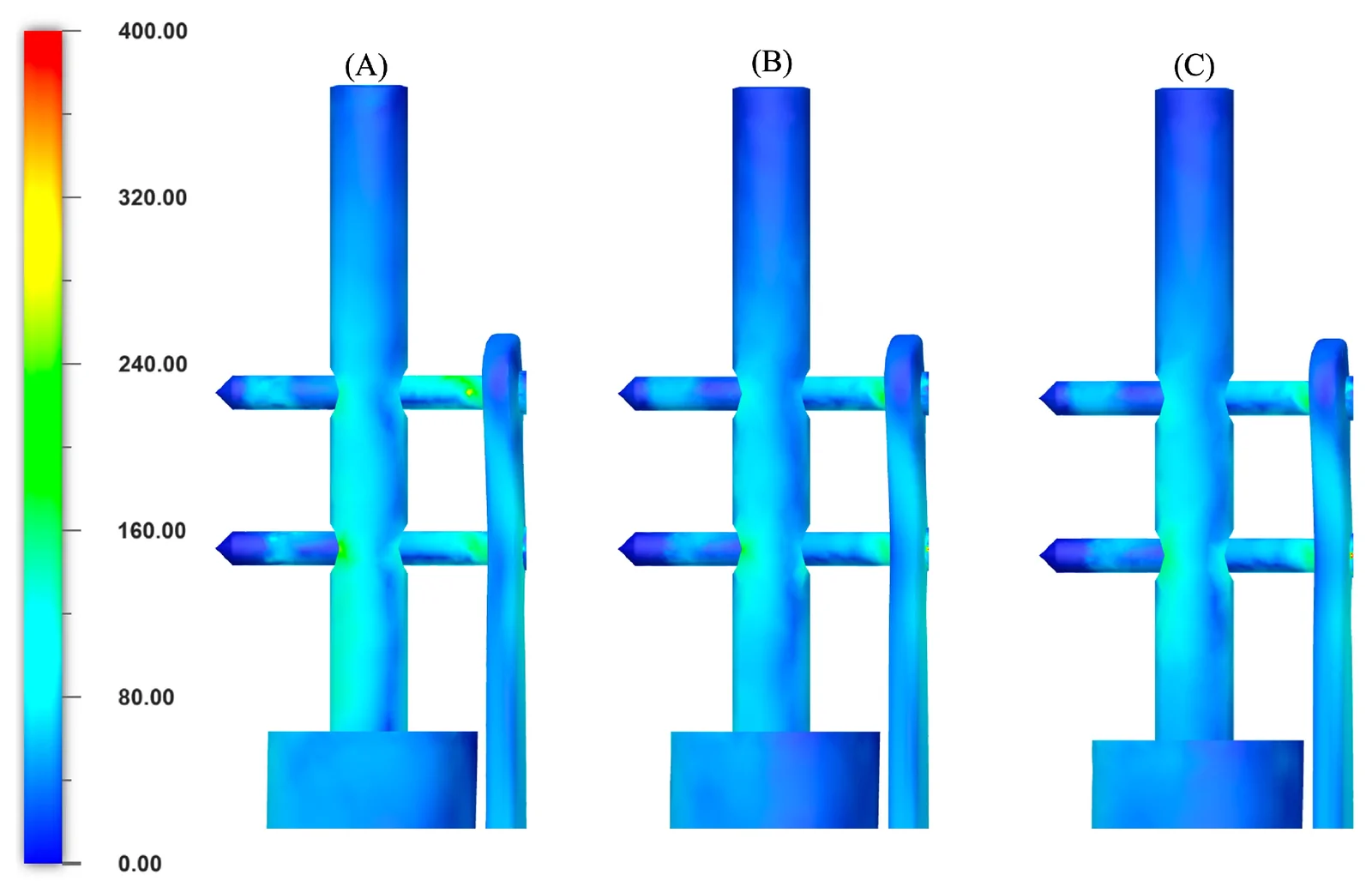
Comparison of maximum stress on the solid proximal intra-medullary stem fixation: in the MI (**A**), MBI-1 (**B**), and MBI-2 (**C**). Scale bar is fixed at 400 MPa to enhance color gradients.

The lack of medial symmetry intensifies stresses in the stem and around screw housings, while bi-cortical screws interactions increase local rigidity, raising stress and failure risk. This hypothesis is corroborated by an alarming value of peak stress of 238.62 MPa in the MI. In both MBI models, $\sigma_{max}$ in the dense stem is reduced to 206.58 MPa in MBI-1 and to 184.30 MPa in MBI-2, due to improved stress redistribution (Figure 5). Additionally, maximum strain in these areas remains around 0.2% in all models. Absence of discontinuities and connecting elements prevents stress concentrations, as well as micro-movements of the structures, allowing for more uniform load distribution. As a conclusion, mono-block implants present a reduced overall stress-shielding effect and a more relaxed stress accumulation, suggesting that they might improve bone loading and distribute loads efficiently. However, $\sigma_{max}$ in the optimized MBI-2 model still stands around 30% of titanium alloy yield strength, confirming the idea that an intra-medullary stem fixation might not be suitable to ensure long-term strength even in mono-block solutions. That is to say, increased stress in the area can trigger sudden failure, with premature separation of the stem from the prosthesis, which will require complex restorative surgical procedures for surgeons and would be demanding for patients to endure.

**Figure 6:**
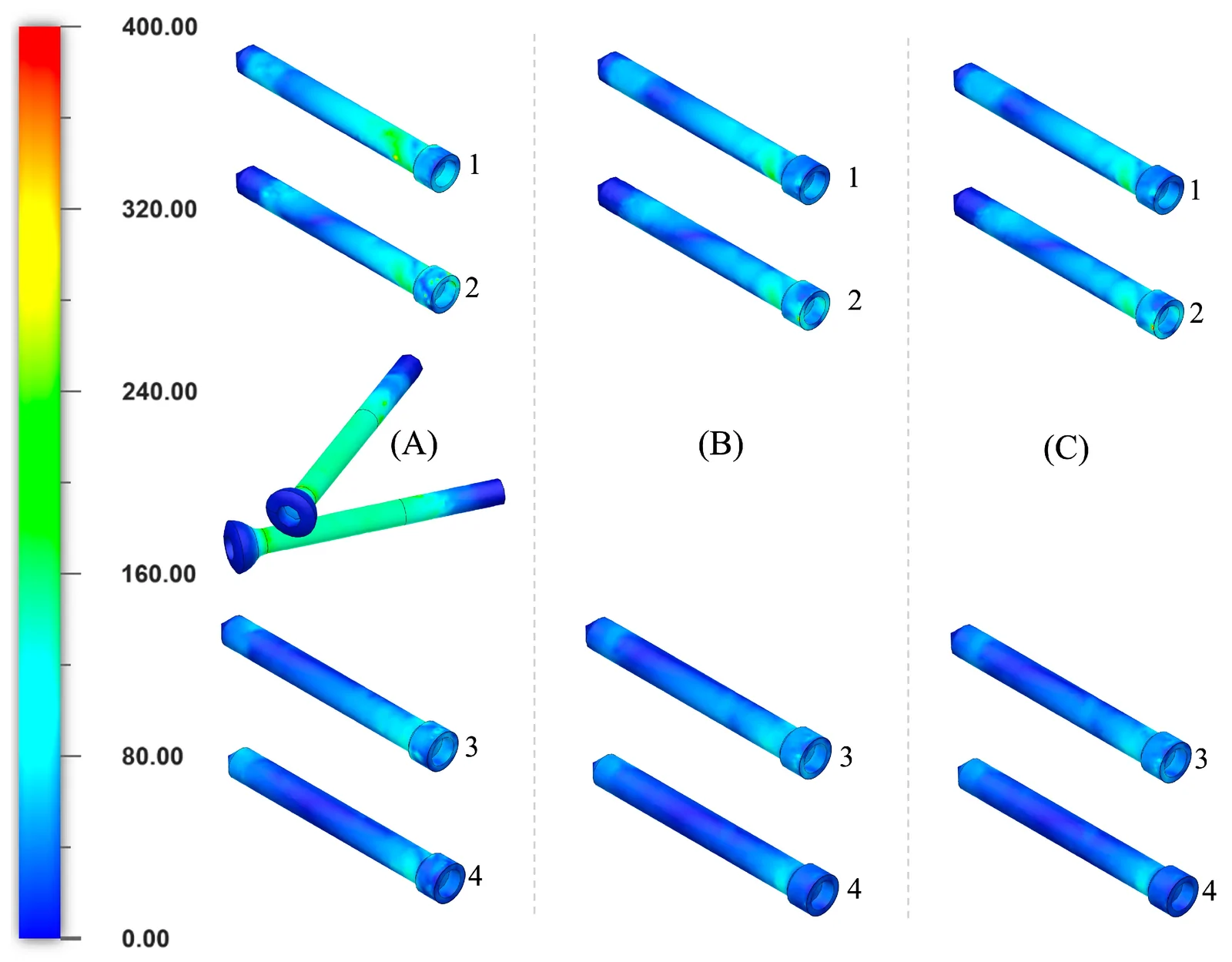
Comparison of maximum stress between screws in the MI (**A**), MBI-1 (**B**), and MBI-2 (**C**). Scale bar is fixed at 400 MPa to enhance color gradients.

In Figure 6, the tensional distribution on locking and linking screws is shown. From details reported in Table 5, fixation screws in the MI appear to experience peak stresses of 470.20 MPa (proximal), near the yield stress limit assumed to be 795 MPa for Ti6AL4V ELI. On the other hand, in the MBI-1 and MBI-2 models, even though the maximum stress on the proximal locking screw No. 2 appears increased up to 673.01 MPa with strains below 1.10%, and all other screws are more evenly loaded with generally reduced stresses from 130.43–360.74 MPa (MI) to 109.21–196.70 MPa (MBI-1), further reduced to 110.48–192.16 MPa (MBI-2). Moreover, the absence of linking screws eliminates the risk of failure of connecting components. Indeed, linking screws in the MI are stressed by combined static loads and 1 kN pre-tension, reaching 329.03 MPa near the heads with strains of 0.3–0.4%.

*Femur bones.* Bone is viscoelastic with high variability, but simulations allow for general conclusions.

**Figure 7:**
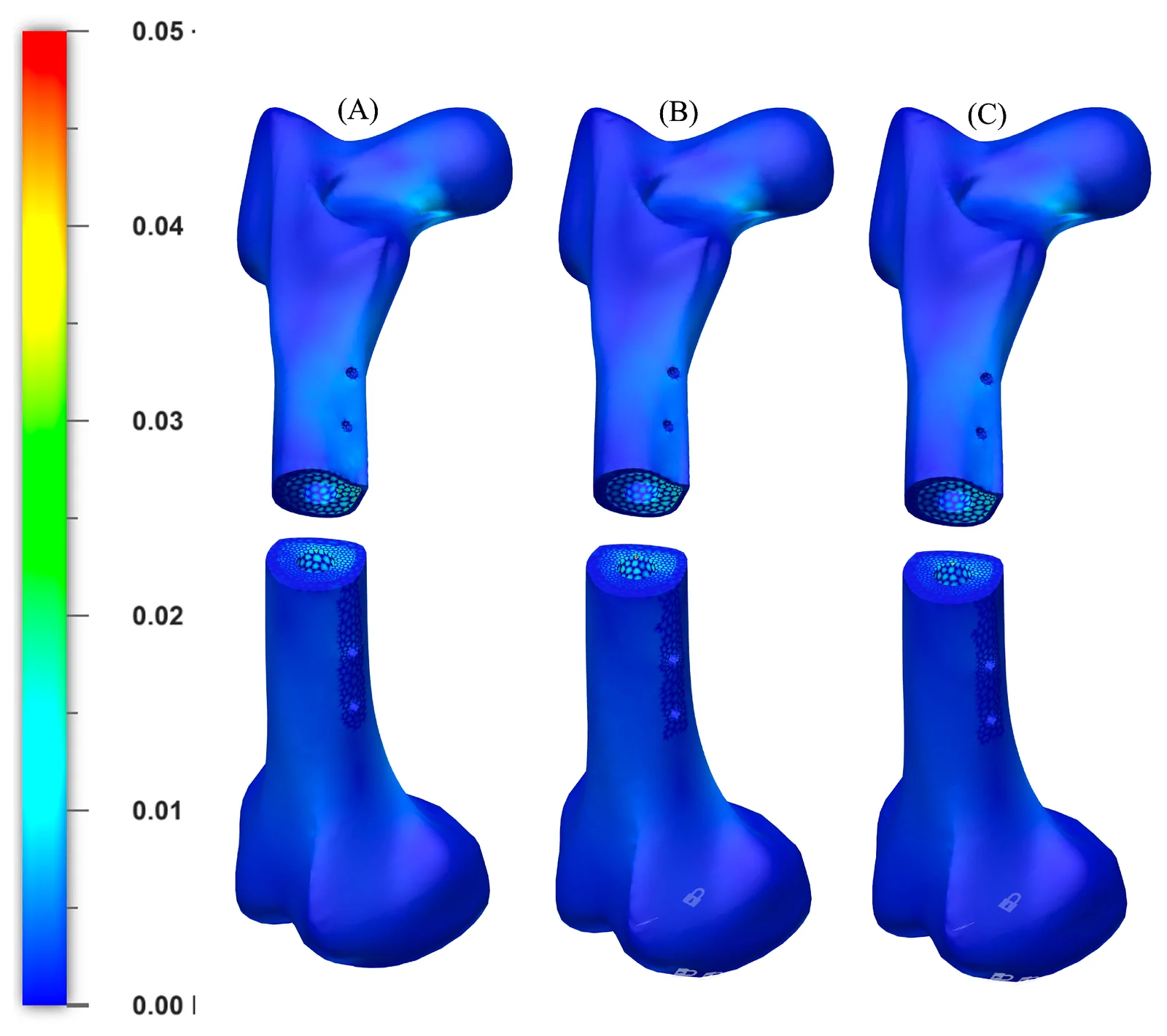
Comparisonof the equivalent strain $\varepsilon_{eq}$ distributions on femoral epiphyses in the MI (**A**) and MBI-1 and -2 (**B**,**C**) models in perspective view.

Volumes in contact with the porous implant show the highest strains, in the range of 4.50–5.10% in the MI and of 4.90–8.30% in the MBIs, which appears to be consistent with the physiological range of strain found in the trabecular bone. As can be easily seen in Figure 7, maximum strains in proximal cancellous bones are similar in both models (∼5%), while distal cancellous strain increases from 4.50% in the MI, to 7.00% and up 8.30% in MBI-1 and MBI-2, respectively (85% increase). Cortical strain decreases from 1.40% in the MI, to 1.20% and 1.00% in MBI-1 and MBI-2, respectively (∼29% reduction). MBIs preserve proximal cortical bone, favoring trabecular stimulation and remodeling by improving elastic solicitation on trabeculae.

Overall, the MBI design effectively redistributes critical stresses, reduces concentrations on screws, maintains physiological bone loading, and supports tissue regeneration, providing improved global stability. However, stress distribution on the stem remains excessive and persistent, keeping the stem critical and susceptible to failure with high probability. The presence of the intra-medullary stem not only represents a starting point for implant failure, but also alters the bone anatomy and elasticity by applying stress-shielding and extremely compressing the bone tissues. Therefore, further investigations of design optimization aimed at improving intramedullary fixation stability are needed.

Comparison of the models suggests that MBIs would be more reliable in terms of mechanical stability and strain-derived osteo-induction of new bone tissue. Under the manufacturing process aspect, 3D printing a whole body composed of porous-dense Ti6Al4V implants would better save feeding powder, which would be wasted in building the supports between the protruding component and the print-bed. As a consequence, a joint assembly would be produced with a less time-consuming production chain, reducing patient waiting time, which is crucial for time-degenerating pathologies such as tumors. Although surgery procedures might appear tougher due to wider distractions needed for MBI implants, patients would benefit from less risky implants that might reduce the likelihood of implant failure, avoiding continuous exposure to reparative surgeries and infections. Regardless of the advantages emerging from the simulations, the proposed results remain ideal and limited to boundary conditions assumed during the setup. Future experimental ex-vivo or in-vivo tests would be needed to confidently state the potential advantages of MBI implants.

## 4. Conclusions

Comparison of the tensile and deformation states between a modular (MI) and a mono-block (MBI) femoral Ti6Al4V implant under static physiological loading was performed using finite element simulations. The mesh convergence study confirmed the stability of the computational models. The spatial distribution of equivalent Von Mises stress was calculated and analyzed for each component of the assemblies. In the MI model, highly critical elements susceptible to failure included the linking screws, locking screws, and distal fixation bridge-plates. A detailed study of these elements in the mono-block configuration showed an overall reduction of stresses on the fixation screws, except for the proximal area, where the stem–screw construct still presented enhanced local rigidity, indicating that the fixation mechanism may require improvement to enhance durability. The increased cortical stress in the MI was attributed to higher local rigidity at the bone/implant interface, potentially leading to bone regression rather than regeneration. In contrast, the MBI was designed without connecting parts or linking screws. Fixation relied on lateral plates locked at both epiphyses. This design significantly outperformed the MI, producing more homogeneous stress and strain distributions and reduced stress concentration on screws and cortical bone. Cortical bone stress in the MBIs reached values within its bearing capacity, whereas cancellous bone deformation remained within the physiological range. Consequently, the MBI system offers improved stability and reliability under static compression, ensuring adequate load-bearing capacity and optimal elasticity, which may promote faster and more effective bone regeneration. On the whole, these findings highlight the potential of the mono-block design to redefine femoral reconstruction strategies. By minimizing stress concentrations and enhancing uniform load transfer, the single-plate MBI implant not only reduces the risk of mechanical failure but also creates a more favorable environment for bone remodeling and long-term implant integration. This innovative approach represents a promising step toward safer, more durable, and biologically harmonious orthopaedic solutions, paving the way for improved patient outcomes and accelerated recovery.

## Figures and Tables

**Table 1:** Materials selected for the computation and their mechanical properties.

Material	Young’s Modulus (GPa)	Poisson’s Ratio
Cortical bone	17.00	0.30
Cancellous bone	0.35	0.25
Ti6Al4V ELI 23	112.40	0.40
Porous Ti6Al4V ELI 23	0.90	0.40

**Table 2:** Materials assignment for bodies in the simulation.

Component	Material
Cortical bone (proximal)	Cortical bone
Cortical bone (distal)	Cortical bone
Cancellous bone (proximal)	Cancellous bone
Cancellous bone (distal)	Cancellous bone
Porous implant (proximal)	Porous Ti6Al4V ELI grade 23
Porous implant (distal)	Porous Ti6Al4V ELI grade 23
Dense implant (distal)	Ti6Al4V ELI grade 23
Dense implant (proximal)	Ti6Al4V ELI grade 23
Screws	Ti6Al4V ELI grade 23

**Table 3:** List of contacts type between interfaces grouped by bodies.

First Component	Second Component	Contact Type
Cortical bone (proximal)	Cancellous bone (proximal)	Bonded
Cortical bone (distal)	Cancellous bone (distal)	Bonded
Porous implant (proximal)	Dense implant (proximal)	Bonded
Porous implant (distal)	Dense implant (distal)	Bonded
Porous implant (proximal)	Cortical bone (proximal)	Separation
Porous implant (proximal)	Cancellous bone (proximal)	Separation
Porous implant (distal)	Cortical bone (distal)	Separation
Porous implant (distal)	Cancellous bone (distal)	Separation
Dense implant (distal)	Cortical bone (distal)	Separation
Dense implant (proximal)	Cortical bone (proximal)	Separation
Dense implant (proximal)	Dense implant (distal)	Rough
Locking screws 1–4	Cancellous bone (proximal)	Bonded
Locking screws 1–4	Cortical bone (distal)	Bonded
Linking screws 1–2	Dense implant (proximal)	Bonded
Linking screws 1–2	Dense implant (distal)	Separation

**Table 4:** Details about the optimal mesh used for the simulations.

Mesh Property	MI	MBI-1	MBI-2
Number of elements	940,238	544,708	572,406
Number of nodes	1,418,259	846,935	885,053
Average element size	3%	3%	3%
Element type	Solid	Solid	Solid
Element order	Parabolic	Parabolic	Parabolic
Scale mesh per part	Yes	Yes	Yes
Existing curved elements	No	No	No
Min. element size (% of avg. size)	5%	5%	5%

**Table 5:** Comparison of maximum Von Mises stress and strain between MI and MBI implants (3% average element size, 5% minimum element size).

Component	MI		MBI-1		MBI-2	
	$\sigma_{max}$	$\varepsilon_{max}$	$\sigma_{max}$	$\varepsilon_{max}$	$\sigma_{max}$	$\varepsilon_{max}$
	(MPa)	(%)	(MPa)	(%)	(MPa)	(%)
Cortical bone (prox.)	174.81	1.40	120.82	1.20	115.21	1.10
Cortical bone (dist.)	60.62	0.60	54.98	0.50	49.32	0.50
Cancellous bone (prox.)	11.11	5.10	10.88	5.10	10.39	4.90
Cancellous bone (dist.)	9.31	4.50	15.72	7.00	18.56	8.30
Dense bridge (dist.)	570.42	0.90	560.45	0.80	547.58	0.90
Dense stem (prox.)	238.62	0.20	206.58	0.20	184.30	0.20
Locking screw 1 (prox.)	360.74	0.50	196.70	0.30	192.16	0.30
Locking screw 2 (prox.)	470.20	0.70	530.79	0.90	673.01	1.10
Locking screw 3 (dist.)	197.26	0.30	124.40	0.20	116.76	0.10
Locking screw 4 (dist.)	130.43	0.20	109.21	0.20	110.48	0.20
Linking screw 1	289.12	0.30	–	–	–	–
Linking screw 2	329.03	0.40	–	–	–	–

## Data Availability

Data will be made available on request.
